# Multi‐Scale Bionic Structure Constructs Biomass Flame‐Retardant Thermal Insulation Foam Material

**DOI:** 10.1002/advs.77015

**Published:** 2026-08-11

**Authors:** Jianming Liao, Lijun Fan, Yunyuan Dong, Xuyu Bao, Xiaobin Chen, Qifu Zheng, Guanqing Zhang, Shen Wang, Wei Chen, Min Zhang, Shuaiming He

**Affiliations:** ^1^ College of Chemical and Material Engineering Quzhou University Quzhou China; ^2^ College of Chemical Engineering Zhejiang University of Technology Hangzhou China; ^3^ School of Pharmaceutical and Chemical Engineering Taizhou University Jiaojiang China; ^4^ State Key Laboratory of Woody Oil Resource Utilization College of Materials and Energy Central South University of Forestry and Technology Changsha China

**Keywords:** biodegradability, cellulose, composites, flame retardancy, thermal insulation

## Abstract

Amid intensifying environmental and energy pressures, sustainable thermal‐insulation materials that also provide effective fire protection are increasingly needed for buildings. Cellulose‐based foams are promising candidates for building‐envelope applications; however, their practical deployment is hindered by limited fire performance, inadequate structural stability, and complex processing. Here, we propose a multiscale biomimetic strategy inspired by mussel adhesion and hierarchical brick‐and‐mortar architectures. Polydopamine is introduced as an interfacial bridging layer to uniformly immobilize bentonite nanosheets within a cellulose network, enabling the fabrication of high‐efficiency flame‐retardant cellulose‐based biomimetic foam (CBF) through aqueous mechanical foaming and ambient‐pressure drying. The resulting CBF exhibits low thermal conductivity alongside improved flame retardancy and environmental compatibility. A cradle‐to‐grave life‐cycle assessment further indicates lower greenhouse‐gas emissions and reduced toxicity‐related impacts than conventional petroleum‐derived foams, while retaining recyclability and biodegradability. Collectively, these results establish a green, scalable route to high‐performance, degradable thermal‐insulation materials for safer and more energy‐efficient buildings.

## Graphical abstract

Inspired by mussel adhesion and hierarchical brick‐and‐mortar architectures, a cellulose‐based biomimetic foam is constructed by anchoring bentonite nanosheets onto a polydopamine‐decorated cellulose nanofiber network. The resulting foam combines low thermal conductivity with intrinsic flame retardancy and full biodegradability, presenting a sustainable, scalable alternative to petroleum‐based insulation materials.

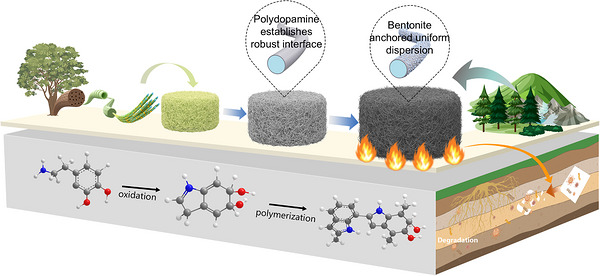

## 1 Introduction

As the climate crisis deepens and concerns over energy security intensify, the buildings and construction sector, responsible for more than one‐third of global energy use and greenhouse‐gas emissions, must accelerate its transition to sustainability. Enhancing the building thermal envelope with insulation foams remains one of the most effective strategies for reducing operational energy demand and associated greenhouse‐gas emissions [1, 2, 3]. Yet progress is limited by two challenges. First, petroleum‐derived plastics impose substantial life‐cycle burdens, including microplastic release, ecotoxicity, and end‐of‐life emissions that can erode net climate benefits [4, 5, 6]. Second, widely used foams such as expanded polystyrene (EPS) and extruded polystyrene (XPS) pose acute fire‐safety risks [7, 8]. Statistics compiled by the International Association of Fire and Rescue Services (CTIF) indicate an average of ∼3.8 million fires per year across reporting countries during 2019–2023, with ∼33.6% originating in buildings [9]. Together, these pressures underscore the need for next‐generation insulation materials that combine high thermal efficiency with intrinsic flame resistance.

To mitigate the environmental and safety liabilities of conventional foams, research has increasingly shifted toward petroleum‐free systems enabled by advances in materials design and microstructural control [10, 11, 12]. Bioplastics such as polylactic acid (PLA) can be processed into microcellular foams with tunable porosity, for example through supercritical CO_2_ foaming [13]. However, large‐scale deployment may introduce additional environmental pressures linked to agricultural production, including water use and inputs of fertilizers and pesticides, potentially weakening their overall sustainability advantage [14, 15]. By contrast, foams assembled from renewable building blocks such as cellulose, chitosan, and gelatin—often reinforced with nanocellulose—offer clear advantages in renewability and potential circularity [16, 17, 18]. Nonetheless, cellulose‐based foams still face barriers to commercialization, including poor interfacial adhesion, energy‐intensive processing, and intrinsic flammability and brittleness [19, 20].

To address structural instability and flammability in cellulose‐based foams, current research increasingly emphasizes molecular‐level bonding strategies. Traditional mechanical processing requires substantial energy input for fiber‐surface activation; moreover, three‐dimensional networks formed solely by hydrogen bonding often collapse because of large inter‐fiber distances and insufficient bond strength [21, 22, 23]. Chemical cross‐linking can increase network integrity, but it often relies on high loadings of flame retardants to counter the inherent combustibility of biopolymers  [24, 25, 26]. These additives can agglomerate because of thermodynamic incompatibility between bio‐derived flame retardants (e.g., phytic acid and lignin) and polymer matrices, thereby degrading microcellular morphology and mechanical performance [27, 28]. Emerging solutions therefore move beyond simple physical blending toward deliberate engineering of interfacial interactions [29, 30]. For example, hydroxyl‐rich biomass building blocks can be leveraged to form dense hydrogen‐bonding networks, while reactive design can covalently incorporate flame‐retardant motifs into polymer backbones to reduce migration and suppress phase separation [31, 32, 33]. Such molecular and interfacial integration can enhance smoke suppression and condensed‐phase char formation without compromising structural coherence, offering a route to foams that are thermally insulating, fire‐safe, and life‐cycle sustainable. In this context, CNF is an ideal structural scaffold because its high aspect ratio, preserved crystallinity, and nanoscale uniformity enable continuous load transfer and char formation, while also stabilizing foam processing. The introduction of polydopamine (PDA) forms a fire‐active interphase, which strengthens adhesion, preserves the porous architecture, and promotes radical scavenging and charring. Together, CNF and PDA offer a minimal yet effective design logic for integrating mechanical robustness, processability, and intrinsic flame resistance in scalable, low‐energy cellulose foam [34, 35, 36, 37].

**FIGURE 1:**
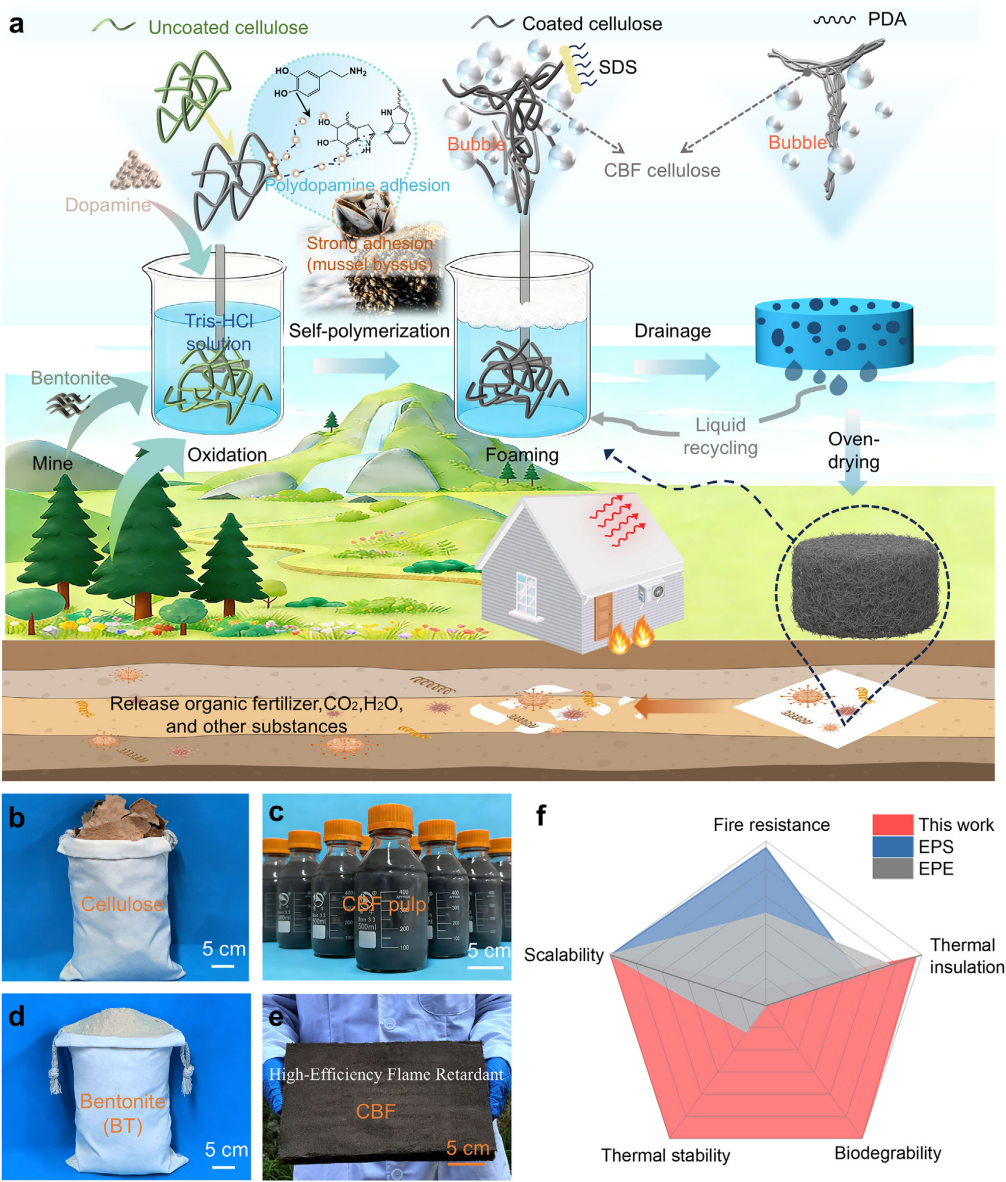
Overview of the design concept and closed‐loop preparation strategy for CBF foam, highlighting its sustainable fabrication from biomass‐derived components for flame‐retardant and thermal insulation applications. (a) Schematic diagram illustrating the preparation and degradation of CBF foam. (b) Cellulose can be extracted from readily available natural resources such as wood, bamboo, cotton, and other plant‐based materials. (c) A large volume of uniform and stable CBF suspension can be easily obtained through mechanical stirring. (d) BT is a clay mineral primarily composed of montmorillonite and is widely found in the natural environment. (e) The CBF foam is lightweight, flame‐retardant, and thermally insulating. (f) Radar chart comparing CBF with conventional insulation materials (EPS and EPE), demonstrating that CBF exhibits superior thermal insulation, mechanical strength, and flame retardancy due to the synergistic effect of BT and dopamine within the foam matrix.

Here, we report a bio‐inspired, multiscale structural strategy that draws on mussel‐inspired adhesion and brick‐and‐mortar architectures. Polydopamine (PDA), via its catechol groups, establishes strong adhesive interfaces within a cellulose nanofiber (CNF) network, enabling robust anchoring and uniform dispersion of bentonite (BT) nanosheets (Figure la). Combined with mechanical foaming and ambient‐pressure drying, this aqueous process yields stable, highly porous foams. Within the solid framework, PDA and BT act synergistically to enhance flame retardancy and promote char formation at elevated temperatures, while the multiscale pore architecture suppresses solid‐state conduction, gas‐phase convection, and radiative heat transfer to deliver high thermal resistance at low density. Importantly, renewable biomass feedstocks can be integrated into established pulp and foam‐processing lines, reducing barriers to industrial scale‐up (Figure 1b–e). The foam's recyclability and biodegradability position it as a sustainable, high‐performance alternative to commercial insulation foams (Figure 1f). Overall, the proposed aqueous, ambient‐pressure processing route offers an innovative pathway with broad application potential for constructing sustainable, high‐performance, flame‐retardant thermal insulation materials.

**FIGURE 2:**
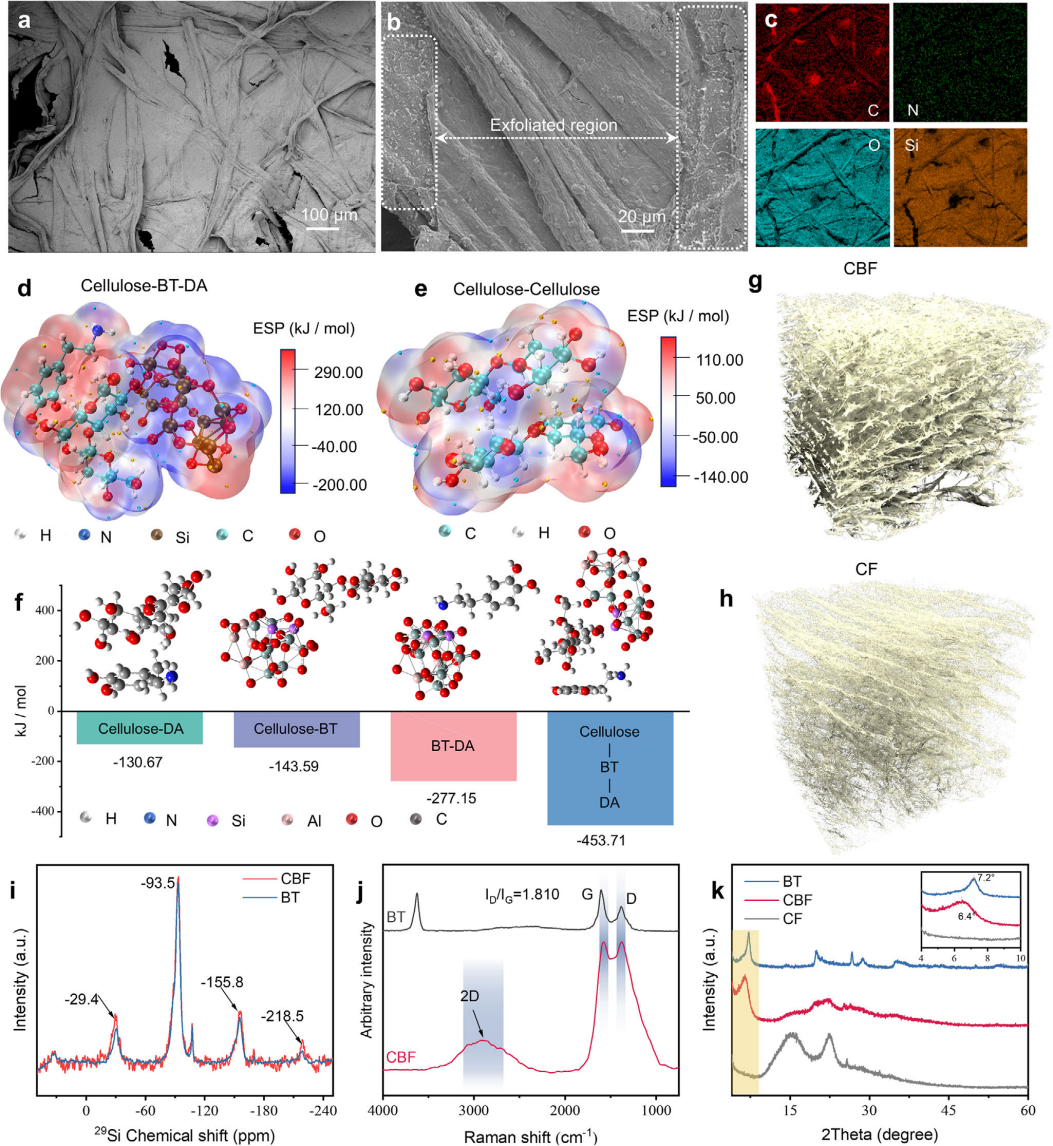
Characterization and computational simulation of CBF. (a) SEM of CBF shows a membrane‐like porous structure from synergistic foaming. (b) High‐mag. SEM reveals dopamine adhesion and the partial exfoliation of BT, enhancing the mechanical‐thermal properties of the composite. (c) EDS confirms uniform Si distribution for consistent flame retardancy. (d) The molecular ESP diagram illustrates enhanced interfacial polarity and bonding in the cellulose‐DA‐BT system. (e) The schematic compares the weakened cellulose interface without additives to the enhanced adhesion in CBF, highlighting the role of dopamine and BT. (f) Binding energy of various binding forms between Cellulose, BT, and DA. (g, h) Micro‐CT reconstructed images visually contrast the more integrated porous network of CBF with the looser structure of plain cellulose foam. (i) Solid‐state NMR indicates successful cellulose/dopamine intercalation into BT. (j) Raman spectra corroborate chemical interactions. (k) XRD (inset 2θ = 3–10°) demonstrates intercalation and hybrid structure formation aiding expansion and flame‐retardant efficiency.

It was found that structural stability across scales, from the molecular to the macroscopic, is markedly enhanced by the synergistic interactions among cellulose, PDA, and BT. After full dispersion of CNF and BT, numerous uniformly distributed heterogeneous nucleation sites were established at fiber interfaces and interstitial voids. Bubble growth at these sites yielded a uniform honeycomb‐like structure (Figures S1 and S2), forming the basis of the three‐dimensional porous architecture. SEM/EDS (Figure 2a–c and Figure S3) further shows BT nanosheets uniformly anchored on PDA‐coated cellulose fibers. In contrast, CBF‐P0 exhibits only sparse BT attachment and significantly lower intensities of Si, Mg, and Al in its EDS spectrum (Figure S4), indicating insufficient BT loading. Such low BT loading inevitably leads to degraded flame retardancy. This confirms that PDA plays an indispensable role as an adhesion promoter to enable effective BT immobilization and multiscale integration. During dopamine self‐oxidation to form PDA, intercalation and surface functionalization at BT interfaces bridge nanoscale building units to microscale fiber assemblies and integrate them into a continuous multiscale architecture (Figure S5), thereby improving overall performance.

The structural evolution mechanism was elucidated by DFT and multiscale characterization. DFT reveals atomic‐scale interfacial interactions (Figure 2d–f; Figures S6 and S7, and Note S1): dopamine preferentially binds to Si–OH sites on BT through hydrogen bonding and electrostatic interactions. The pristine cellulose–BT system shows a binding energy of −143.59 kJ mol^−^^1^, whereas the cellulose–dopamine, BT–dopamine, and ternary systems reach −130.67, −277.15, and −453.71 kJ mol^−^^1^, respectively, indicating that dopamine serves as an effective molecular bridge that enhances interfacial compatibility and stress transfer. As shown in Figure 2g,h and Figures S8 and S9, CBF exhibits a denser architecture with slight boundary densification and no obvious collapse, whereas the cellulose foams (CF) contain large pores and weak junctions; its finer skeleton and sparse connectivity make it susceptible to localized collapse. This contrast accounts for the superior mechanical and flame‐retardant performance of CBF. Solid‐state 29Si NMR (Figure 2i) supports this assignment: the presence and strengthening of Q^3^ resonances characteristic of Si‐OH and the emergence of Q^2^ environments are consistent with the formation of an organic–inorganic interpenetrating network at the interface, while the persistence of Q^4^ signals confirms an intact silicate framework [38]. Raman spectroscopy (Figure 2j and Figure S10) further evidences reinforced interfaces: the I_D_/I_G_ ratio increases from 0.575 to 1.810 in CBF, suggesting nitrogen‐rich graphitized domains and controllable defect sites that act as efficient energy‐dissipation centers [39, 40].

At the molecular‐interface level, FTIR (Figure S11) shows a redshift and broadening of the O–H band, confirming hydrogen‐bond network formation. Solid‐state ^13^C NMR (Figure S12) shows preferential attenuation of the amorphous C4 signal (∼88.9 ppm), indicating chain rigidification induced by cross‐linking. XPS (Figures S13 and S14) further reveals enhanced C═O/aromatic C signals, supporting PDA incorporation; weakened C–O contributions, consistent with hydrogen‐bond participation; and broadened Si 2p features, evidencing PDA–BT interfacial interactions. Collectively, these results corroborate that dopamine acts as a molecular bridge, anchoring at cellulose and BT Si–OH sites and driving reorganization of the inorganic phase [41, 42, 43, 44]. Consistent with these findings, XRD patterns show significant broadening (Figure 2k), weakening, and low‐angle shifts of BT diffraction peaks, indicating intercalation‐driven expansion of interlayer spacing and partial delamination [45]. The resulting uniformly dispersed state and dense interpenetrating network within the matrix effectively avoid the collapse of the macroscopic structure. Altogether, multiscale chemical coupling and structural optimization provide a synergistic mechanism: an intact Si‐O framework (Q^4^) and the interpenetrating network confer high stiffness and thermal stability, while graphite‐like interfacial domains and defects dissipate energy through local structural reorganization. Guided by this “rigid framework support‐flexible interfacial dissipation” synergy, CBF achieves simultaneous improvements in strength and toughness. As shown by the stress–strain curves (Figures S15 and S16), CBF‐4 exhibits the strongest reinforcement, with a compressive strength of 123.6 kPa, approximately three times that of unmodified CF, and a specific compressive modulus of 4.19 MPa cm^3^ g^−1^, approximately 2.6 times that of CF and CBF‐0. This improved load‐bearing capacity and deformation resistance at low density are mainly attributed to dopamine‐mediated adhesion, which promotes strong interfacial integration between bentonite nanosheets and cellulose fibers. The resulting three‐dimensional reinforcing network stabilizes the pore walls and improves load transfer efficiency, demonstrating that moderate synergistic modification with bentonite and dopamine effectively enhances the compressive performance of cellulose‐based foams.

**FIGURE 3:**
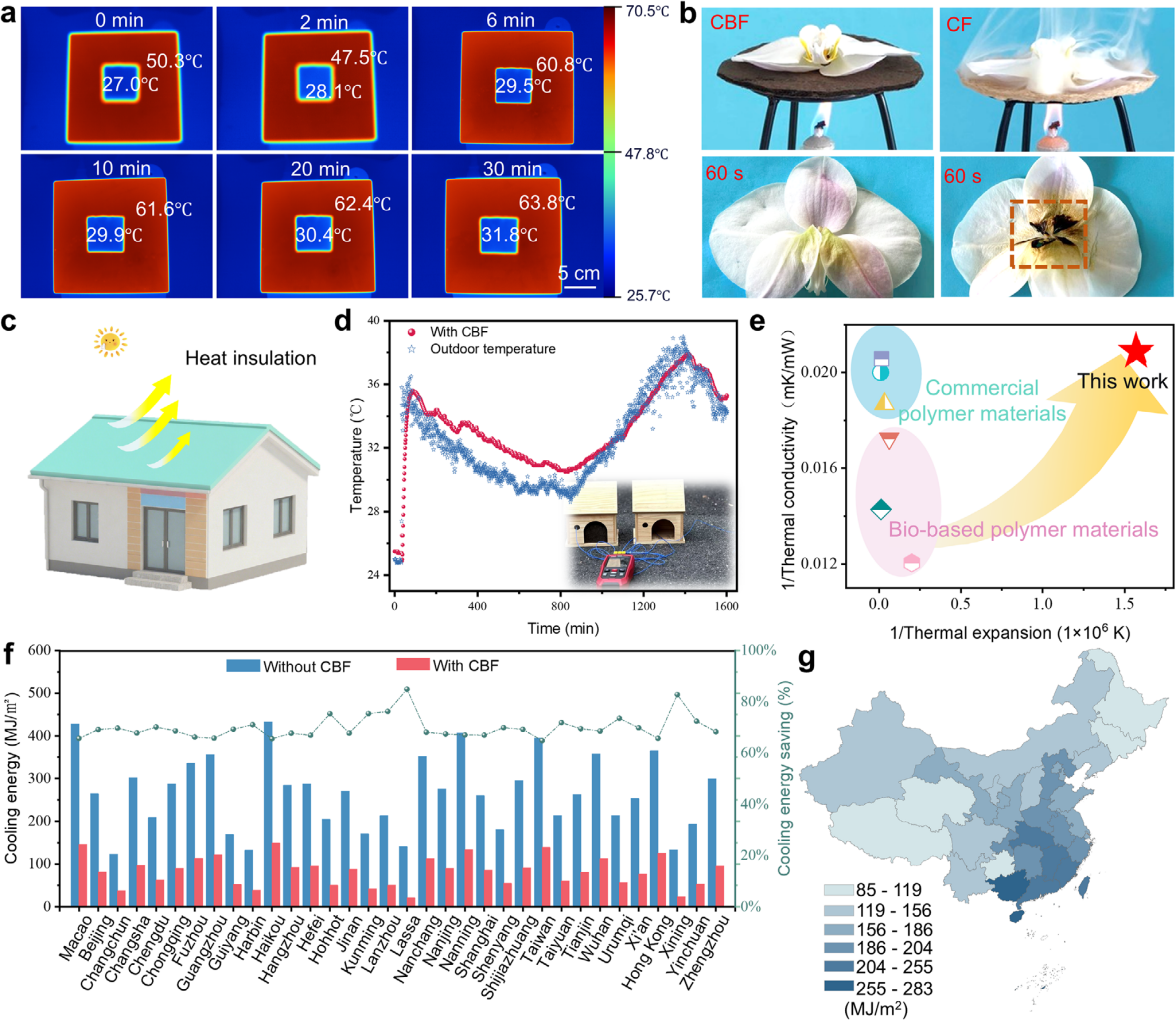
Thermal insulation performance of CBF. (a) Infrared thermal image showing the surface temperature distribution of CBF, highlighting its ability to maintain lower temperatures under thermal exposure owing to its porous, BT‐enhanced structure. (b) Comparison of thermal insulation performance between CBF and plain cellulose foam, confirming the superior thermal resistance achieved through dopamine‐mediated bonding and BT incorporation. (c) Schematic diagram illustrating the building insulation simulation using CBF foam, demonstrating its effectiveness as a bio‐based insulation layer in wall assemblies. (d) Recorded temperature profiles from the building insulation simulation, indicating that CBF significantly reduces heat transfer compared with outdoor temperature. (e) Comparative analysis of thermal conductivity and coefficient of thermal expansion data with other commercial polymer foam materials and bio‐based materials shows that CBF has excellent thermal stability. (f) Simulated annual cooling energy consumption of building models in different cities, illustrating the potential energy savings enabled by the high‐efficiency insulating CBF foam. (g) Estimated annual energy savings across various climate zones in China, demonstrating the broad applicability and environmental benefits of CBF foam in reducing building energy consumption.

Thermal insulation layers are critical to building energy efficiency [46]. However, many commonly used organic insulating materials impose environmental burdens during production and end‐of‐life, and their intrinsic fire performance is often inadequate; upon ignition, they can generate toxic smoke and melt dripping, elevating safety risks [47]. Hence, developing inorganic and bio‐based insulating materials that combine high fire safety with environmental compatibility is of practical importance. In this study, CBF was engineered via a multiscale bioinspired structural design, targeting low thermal conductivity alongside high safety. To evaluate through‐thickness heat transfer, CBF specimens were placed on a hot plate (Figure 3a), and the evolution of the top‐surface temperature was monitored by infrared thermography. As heating progressed, the top‐surface temperature rose only modestly; after 30 min, it reached 31.8°C, indicating strongly suppressed heat penetration and excellent thermal insulation. Consistent with this observation, the temperature difference (ΔT) between the top surface and the plate remained at approximately 30°C (Figure S17). To further illustrate the insulation performance, a simple qualitative test was conducted: flower petals were placed on the surfaces of cellulose foam and CBF, and their thermal response was recorded. As shown in Figure 3b and Figure S18, petals on cellulose foam exhibited rapid dehydration and charring within 60 s, whereas petals on CBF retained their fresh appearance and original morphology over the same duration, highlighting the material's effective thermal barrier function. Notably, CBF maintains excellent dimensional stability at elevated temperatures. After exposure to 200°C for 5 min, common commercial plastics—polypropylene (PP) and polyethylene (PE)—softened and deformed, whereas CBF exhibited negligible change. Its coefficient of thermal expansion (CTE) is 0.63 × 10^−^^6^ K^−^^1^, substantially lower than those of the above plastics, underscoring its high‐temperature stability (Figure S19). As illustrated in Figure 3c, CBF can be integrated into building envelopes such as exterior walls and roofs, demonstrating its potential as a sustainable, high‐performance insulation solution.

To evaluate performance under realistic conditions, outdoor monitoring was conducted in Quzhou, China, on 28–29 August 2025 (Figure 3d). The thermal conductivity and coefficient of thermal expansion of CBF were compared with those of representative polymer‐based materials (Figure 3e; see also Table S1). Despite pronounced fluctuations in ambient conditions, the temperature inside the small wooden cabin insulated with CBF remained comparatively stable, consistent with its strong thermal‐insulation performance. CBF also combines low thermal conductivity with low density (Table S2 and Figure S20), highlighting its potential as a lightweight thermal‐insulation material. To mitigate the inherent hygroscopicity of cellulose‐based materials, a simple post‐treatment using polydimethylsiloxane (PDMS) was applied via dip‐coating. This treatment rendered the material hydrophobic, resulting in a water contact angle of 103° and markedly improved moisture resistance (Figure S21), thereby supporting its suitability for practical applications.

Energy consumption simulations were conducted with EnergyPlus (see Note S2 and Figure S22) to compare annual cooling loads for buildings with and without CBF infill. The baseline exterior wall was defined as a conventional residential wall representative of the hot‐summer and warm‐winter climate zone in China, consisting of 20 mm cement mortar, 200 mm masonry block, and 20 mm cement mortar, with a heat transfer coefficient of U = 0.857 W m^−^^2^ K^−^^1^ based on the Design Standard for Energy Efficiency of Public Buildings GB 50189–2015. In the EnergyPlus model, the CBF‐filled wall assembly was assigned a U‐value of 0.549 W m^−^^2^ K^−^^1^ based on the effective thermal properties of the complete wall system. The simulations were run under an identical building model, operating schedule, indoor temperature settings, and climatic boundary conditions. The results (Figure 3f) show substantial reductions in cooling energy across all modeled cities, with a mean savings of 69.95% under the specified assumptions. Analysis by climate zone (Figure 3g) indicates that the energy‐saving potential of CBF is more pronounced in tropical and subtropical regions (Tables S3 and S4). These simulation trends align with experimental observations, supporting the superior thermal insulation performance of CBF and its promising potential for building energy conservation.

**FIGURE 4:**
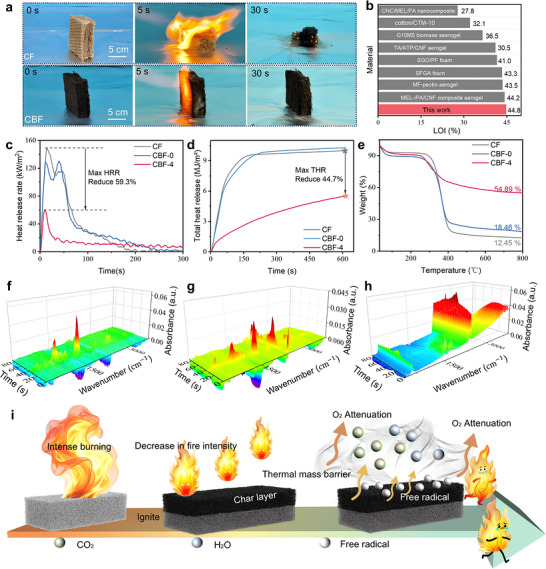
Evaluation of flame‐retardant performance of CBF. (a) Combustion behavior of CF, showing rapid flame spread and complete consumption. CBF‐4 during burning, exhibiting self‐extinguishing behavior and a stable char layer formed by the BT‐dopamine synergy. (b) The Limiting Oxygen Index (LOI) of CBF‐4 compared with other flame‐retardant materials, confirming its enhanced resistance to ignition [49, 50, 51, 52, 53, 54, 55, 56]. (c) Heat‐release rate (HRR) curves from cone calorimetry: CBF‐4 shows a significantly lower peak than CF and CBF‐0, indicating effective flame suppression. (d) Total heat release (THR) is lowest for CBF‐4, demonstrating reduced fire load. (e) TGA indicates CBF‐4's superior thermal stability and char residue due to BT‐dopamine synergy. (f–h) 3D TG‐IR spectra of CF, CBF‐0, and CBF‐4, illustrating that CBF‐4 releases fewer flammable volatiles during pyrolysis. (i) Schematic of the flame‐retardant mechanism in CBF‐4. BT forms a barrier, DA promotes charring, and the char traps free radicals while releasing non‐flammable gases to dilute oxygen, synergistically suppressing combustion.

In energy‐efficient buildings, the fire resistance of insulation is critical for safety. Under exposure to a ∼1300°C flame, neat cellulose ignited rapidly and was consumed within 30 s, whereas CBF‐4 exhibited only surface carbonization after 30 s, self‐extinguished rapidly upon flame removal, and retained its structural integrity (Figure 4a; Figure S23 and Videos S1–S3). UL‐94 vertical tests further show that CBF‐4 achieves a V‐0 rating, and its limiting oxygen index (LOI) is 44.8%, outperforming most reported flame‐retardant insulating materials (Figure 4b and Figures S24 and S25). Cone calorimetry quantifies this behavior: the peak heat release rate (PHRR) of CBF‐4 is 60.3 kW·m^−^^2^—a 59.3% reduction relative to cellulose foam (148.9 kW·m^−^^2^)—, and the total heat release (THR) decreases by 44.7%. The CO_2_ release rate and effective heat of combustion (EHC) also decrease, indicating suppressed combustion intensity (Figure 4c,d and Figures S26 and S27) [48]. Combined with the high residual char yield of 54.9% from thermogravimetric analysis (Figure 4e and Figure S28), these results suggest a predominantly condensed‐phase flame‐retardant mechanism, with secondary gas‐phase contributions.

Thermogravimetry‐FTIR (TG‐FTIR) was used to monitor volatile species during thermal degradation (Figure 4f–h and Figure S29). Neat cellulose foam rapidly evolves oxygenated organics, with pronounced gas‐phase bands attributable to carbonyl groups (∼1700–1800 cm^−^^1^), ethers/C‐O‐C (∼1000–1150 cm^−^^1^), CO_2_ (∼2350 cm^−^^1^), and H_2_O (3400–3700 cm^−^^1^), consistent with fragmentation into low‐molecular‐weight volatiles [57]. By contrast, in CBF‐4, PDA establishes a stabilizing interfacial layer on the fibers that promotes dehydration and cross‐linking, diverting the pyrolysis pathway toward char formation. The formation of this stabilizing PDA interface is confirmed by XPS analysis (Figure S30). The C 1s spectrum clearly shows the emergence of a characteristic C═N peak from PDA, while the O 1s spectrum exhibits a marked increase in the relative intensity of the C‐O‐C/C‐O‐H component, indicating the reinforcement of the interfacial network through enhanced hydrogen bonding and possible cross‐linking. Together with the layered BT architecture and the hydrogen‐bond network between PDA and cellulose, this multiscale barrier increases tortuosity for heat and mass transport, delaying volatilization and attenuating the intensity of the principal gas‐evolution peak, which shifts to higher temperatures. PDA also facilitates the formation of an aromatic‐rich carbonaceous char that interlocks with lamellar silicates, strengthening the integrity of the protective layer; additionally, phenolic/quinone moieties can scavenge gas‐phase radicals, mitigating flame propagation (Figure 4i and Figures S31–S33). Overall, the organic–inorganic synergy in CBF‐4 enhances thermal stability and flame retardancy while preserving the porous architecture, underscoring its application potential.

**FIGURE 5:**
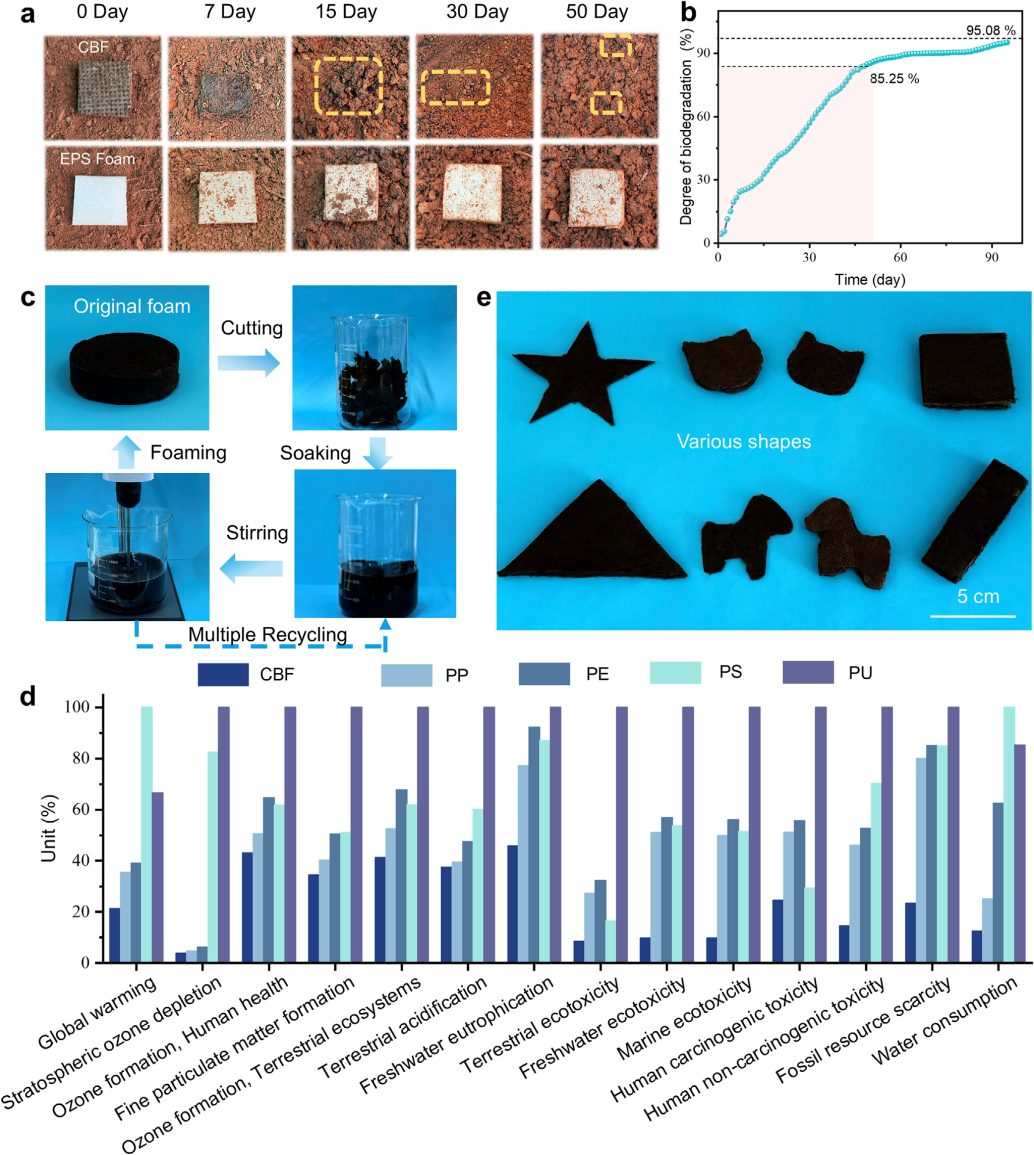
Environmental Impact Assessment of CBF. (a) Soil‐burial biodegradation tests comparing CBF with polystyrene (PS) show rapid decomposition of the cellulose‐based CBF foam, whereas PS remains largely persistent, consistent with CBF's bio‐derived porous architecture. (b) The cellulose biodegradation curve further depicts the enzymatic degradation profile of the pulp substrate used in CBF, supporting its compostability. (c) Recyclability tests demonstrate that CBF can be reprocessed multiple times with minimal loss of thermal insulation and mechanical performance, enabled by a dopamine‐BT cross‐linked network. (d) A comparative life‐cycle impact assessment of CBF and petrochemical plastics (PP, PE, PS, and PU) highlights CBF's lower carbon footprint and reduced ecotoxicity, attributable to its bio‐based composition. (e) Processing CBF into multiple geometries confirms its adaptability to diverse insulation applications while maintaining flame‐retardant and thermally resistant performance derived from the synergistic interactions among BT, dopamine, and nanocellulose.

For practical deployment, insulation foams must exhibit high performance, biodegradability, and overall environmental sustainability. We first assessed the weatherability of CBF foam via accelerated UV aging following ASTM G155 (xenon arc, daylight filter, 0.35 W/(m^2^·nm) at 340 nm, 60°C, 168 h). Daily photographic documentation revealed no significant discoloration or structural degradation over the 7 days of exposure (Figure S34), confirming the UV‐shielding effectiveness of PDA. We then evaluated the biodegradability of CBF foam under soil burial. Square specimens (5 cm × 5 cm; thicknesses specified in the Methods) were buried in soil, and their macroscopic morphology was documented at regular intervals (Figure 5a). Over time, the cellulose matrix progressively decomposed, leading to disintegration of the foam architecture; after approximately 50 days, specimens had lost structural integrity. BT, an inorganic mineral, is not biodegradable but is generally regarded as environmentally benign (Note S3); any residual mineral particulates are expected to impose minimal environmental impact. In complementary composting tests (Note S4), mature municipal compost (three months) was used to quantify cellulose degradation via CO_2_ evolution, with thin‐layer chromatography (TLC) grade cellulose serving as a reference. A biodegradation extent of 85.25% was observed after 50 days (Figure 5b), evidencing the microbial degradability of the cellulose component. These results indicate that CBF foam can degrade rapidly under natural soil conditions, thereby reducing its end‐of‐life burden on the environment. Moreover, CBF foam is readily recyclable and reusable (Figure 5c); a simple recovery and reprocessing workflow enables material recycling, facilitating safer disposal and resource circularity.

In practical applications, insulation foams must deliver high performance while minimizing life‐cycle burdens and enabling responsible end‐of‐life management [58]. Developing sustainable materials and reducing energy use across the product life cycle are essential for long‐term environmental benefits. To systematically evaluate environmental performance, we conducted a cradle‐to‐grave life cycle assessment (LCA) comparing CBF with foams based on polypropylene (PP), polyethylene (PE), polystyrene (PS), and polyurethane (PU) (Note S5). The system boundary covers raw material extraction, manufacturing, and end‐of‐life. Cellulose substitution lowers impacts in most categories, with CBF outperforming commercial foams in 14 indicators (Figure 5d and Table S5). However, energy data are from lab‐scale batch equipment—not industrial scale—and upscaling faces challenges in dispersion uniformity and dopamine cost, which can be addressed by continuous foaming, low‐energy drying, and alternative modifiers. On a functional‐unit basis, CBF's global warming potential is 2.40 kg CO_2_ eq, 39.8%, 45.6%, 78.7%, and 68.0% lower than PP, PE, PS, and PU foams, respectively (full results in Table S6). Because the proposed fabrication strategy is compatible with diverse biobased feedstocks, sourcing cellulose with lower upstream burdens (e.g., bamboo or recovered waste paper; Figure S35) can further improve the sustainability profile. Overall, the material decreases dependence on fossil and mineral resources across the production and use phases and mitigates impacts associated with ecosystem quality and human health. In addition, the foam's customizable form factor (Figure 5e) enables precise design and manufacturing tailored to application needs, which can reduce processing scrap and resource consumption and enhance applicability within sustainable materials systems.

## 2 Conclusions

This study establishes a scalable fabrication route for a CBF through multiscale biomimetic design. By introducing PDA as an interfacial bridge and coupling mechanical foaming with constant‐pressure drying, we achieve uniform immobilization of bentonite (BT) nanosheets within a cellulose network. Relative to a neat cellulose foam, CBF delivers substantially improved thermal insulation, and the thermal conductivity is as low as 48.8 m W/m K. The foam also exhibits strong fire performance (LOI = 44.8% and a UL‐94 V‐0 rating). Upon combustion, CBF forms a dense protective char layer, resulting in a high char residue (54.9%) and pronounced suppression of fire intensity, as reflected by reductions in peak heat release rate (PHRR) and total heat release (THR) of 59.3% and 44.7%, respectively. In addition, CBF shows outstanding dimensional stability, with a low coefficient of thermal expansion (CTE) of 0.63 × 10^−^^6^ K^−^^1^. Based on the building energy consumption simulation using EnergyPlus, it is further shown that the building adopting CBF can achieve an energy saving rate of 69.95%. Owing to the elimination of petroleum‐based raw materials, energy‐intensive processes, and toxic flame retardants, CBF surpasses traditional EPS/XPS foams in resource efficiency, fire safety, and environmental performance. Life cycle assessment (LCA) further confirms substantial reductions across 14 key environmental indicators, particularly in carbon footprint and ecological toxicity. The material achieves 85.25% biodegradation in soil within 50 days, underscoring its recyclability and sustainability. Collectively, these attributes position CBF as a promising alternative for enhancing building energy conservation and fire safety.

## 3 Experimental Section

### 3.1 Materials

The unbleached softwood pulp was provided by Xianhe Co., Ltd. Sodium dodecyl sulfate (SDS) was supplied by Tianjin Fuchen Chemical Reagent Co., Ltd., with a very high analytical purity. Dopamine hydrochloride (DA) and Tris(Hydroxymethyl)Aminomethane Hydrochloride (Tris‐HCl) were purchased from Shanghai Aladdin Biochemical Technology Co., Ltd. Sodium‐based montmorillonite (BT) was provided by Shanlinshi Yuxin Mining Products Co., Ltd. In Guzhang County, with a purity of 90%. Cellulose nanofibers (CNF) were provided by Zhejiang Jinjiaohao Green Nanomaterials Co., Ltd. Polydimethylsiloxane (PDMS) was purchased from Shanghai Aladdin Biochemical Technology Co., Ltd.

### 3.2 Pretreatment of Wood Pulp

A systematic pre‐treatment was carried out on the raw fibers. First, the fibers were immersed in deionized water at room temperature for 4 h to fully swell and soften them. After the soaking process, the fiber slurry was mechanically disintegrated to initially separate the fiber bundles. Subsequently, the fibers were treated with a pulping machine (IMT Valier type pulping machine) at a specific speed for a certain period of time to further fibrillate the fibers.

After the above treatment, the fiber morphology was observed using an optical microscope (as shown in Figures S36 and S37). Subsequently, the treated slurry was centrifuged and dehydrated to remove most of the free water between the fibers. The moisture content of the fibers after centrifugation was precisely determined using a moisture analyzer (Ohaus instrument). Finally, the free volume of the pretreated fiber slurry was measured according to the standard method (in accordance with ISO 5267‐2:2001 standard), and the value was 440 mL CSF. The treated fibers were stored in a refrigerator at 4°C for the next experiment.

### 3.3 Preparation Process of CBF

A series of cellulose‐based biomimetic foams (designated CBF‐x, x = 0–4) with varying bentonite (BT) contents were prepared by adjusting the BT‐to‐cellulose ratio. First, 20 g of mechanically pretreated unbleached coniferous wood pulp was mixed with a dopamine solution (10 wt.% relative to dry fibers). The pH was adjusted to 8.5 using tris(hydroxymethyl)aminomethane hydrochloride, followed by stirring at room temperature for 12 h. The reacted mixture was then transferred to a disintegrator, where cellulose nanofiber (CNF, 6 wt.% relative to dry fibers) and BT powder (0, 25, 50, 75, or 100 wt.% relative to dry fibers) were added. The pulp concentration was adjusted to 2 wt.%, after which sodium dodecyl sulfate (SDS) was introduced, and the suspension was stirred at high speed for 1 min. Once the wet foam volume stabilized, it was cast into a custom polytetrafluoroethylene mold, and excess water was removed by gravity filtration. The mold was then dried at 60°C for 6 h to obtain the final foams. For comparison, plain cellulose foams without BT and DA were also prepared using the same procedure and designated as cellulose foams (CF). Additionally, to evaluate the specific role of dopamine, another control foam (designated CBF‐P0) was prepared following the same procedure, using 100 wt.% BT in the absence of dopamine.

### 3.4 Characterizations

The morphology and structure of the CBF foam were characterized using a scanning electron microscope (Hitachi SU‐70). The chemical composition of the samples was characterized by Fourier Transform Infrared Spectroscopy (FT‐IR), x‐ray photoelectron spectroscopy (XPS), and the German Bruker Avance Neo 400WB solid‐state nuclear magnetic resonance. The x‐ray diffraction patterns of the CF, BT, and the CBF in the diffraction angle (2θ) range from 3 to 60° at a scanning speed of 5° min^−1^ were recorded via a Siemens D5000 x‐ray diffractometer (Siemens, Germany). The thermal gravimetric analysis (TGA) was performed using the Netzsch STA449F3 type thermogravimetric analyzer under nitrogen at a heating rate of 10°C/min from 40°C to 800°C. The TGA‐IR thermal gravimetric‐infrared combination was used under nitrogen at a gas flow rate of 40 mL/min and a heating rate of 10°C/min from 40°C to 800°C. The interaction relationships, hydrogen bond distribution, and hydrogen bond strength of each component in CBF were simulated using density functional theory (DFT) (see Note S1 for details).

The thermal conductivity of the dried foam was measured using the Hot Disk TPS2500 thermal conductivity instrument at ambient temperature. A 20 mm thick CBF sample was placed inside a small wooden house (12 cm × 13.5 cm × 15.7 cm). Outdoor temperature changes were monitored from August 28th, 2025 (weather conditions: sunny 27°C–39°C) in the afternoon to August 29th, 2025 (weather conditions: cloudy 28°C–38°C) in Quzhou (China). The real‐time temperature of the sample was recorded by placing the thermocouple thermometer probe inside the house and outdoors, combined with a multi‐channel data recorder. Infrared thermography was captured by an infrared thermal imager (FOTRIC 285s). The thermal expansion coefficient of the foam was determined using a thermal mechanical analyzer (TA TMA Q400EM) (test temperature range: 200°C–50°C). The thermal stability of the sample was analyzed by monitoring the morphological changes of the material under high temperature by placing the sample in a pre‐set temperature oven. The cooling energy consumption of the building model was numerically simulated using EnergyPlus (Version 9.4) software (see Note S2 for details). Foam samples with a size of 100 × 10 × 10 mm^3^ were selected, and the limit oxygen index (LOI) was measured according to the GB 5454‐1985 standard. According to the requirements of ASTM D3801‐10, horizontal and vertical combustion tests were conducted on the foam sample with a size of 100.0 × 10.0 × 20.0 mm^3^ in the UL‐94 flammability tester (British Fire Testing Technology Co. Ltd.) (size: 100.0 × 10.0 × 20.0 mm^3^). The combustion performance of the foam sample with a size of 100.0 × 100.0 × 5.0 mm^3^ was evaluated according to ISO5660 (external heat flux density = 35 kW m^−^^2^) using a cone calorimeter (British FTT Fire Testing Technology).

The surface morphology and surface properties of nanocellulose were observed using an atomic force microscope (Bruker Dimension ICON, AFM). Compression tests were conducted using an INSTRON 5565 material testing machine with a compression rate of 10 millimeters per minute. The test ended when the compression strain reached 80% of the original height, and the corresponding stress–strain curve was plotted. The sample was cut into a cubic size of 45 mm × 45 mm × 20 mm. The sample was firmly fixed on the upper and lower test plates using double‐sided tape (3 M brand). A pressure of 20 Newtons was applied for 30 s to ensure firm adhesion of the double‐sided tape. Subsequently, the upper and lower test plates were gradually separated, and the sample layer was stretched apart until the interlayer bonding strength dropped to 0. At this point, the test stopped.

PP and CBF foam were made into samples of 50.0 × 50.0 × 50.0 mm^3^ for soil biodegradability testing [59]. These specimens were then buried 5 cm deep in natural soil. For observation purposes, specimens were periodically retrieved and recorded. Life Cycle Assessment (LCA) analysis was performed using Simapro 10.1 (details in Note S5). To evaluate the long‐term environmental durability of the CBF foams, we conducted an accelerated UV aging test following the ASTM G155 standard. The specimens were exposed to a xenon arc lamp with a daylight filter at an irradiance of 0.35 W/(m^2^·nm) (at 340 nm) and a black panel temperature of 60°C for a total of 168 h (7 days). Samples were collected at 0, 24, 48, 72, 96, 120, 144, and 168 h for photographic documentation of macroscopic appearance change.

## Author Contributions

J. Liao, L. Fan, and Y. Dong designed the experiments. J. Liao, L. Fan, Y. Dong, X. Bao, W. Chen, and X. Chen carried out material preparation and characterization experiments. X. Chen performed data analysis and supervised the project. Q. Zheng, G. Zhang, S. Wang, and M. Zhang contributed to data interpretation and technical support. S. He conducted simulations and provided methodological guidance. J. Liao, L. Fan, and Y. Dong wrote the manuscript. X. Chen, M. Zhang, and S. He provided financial support and revised the manuscript. All authors commented on the final manuscript.

## Conflicts of Interest

The authors declare no conflicts of interest.

## Supporting information




**Supporting File 1**: advs77015‐sup‐0001‐SuppMat.docx.

**Supporting File 2**: advs77015‐sup‐0002‐VideoS1‐S3.zip.

## Data Availability

The data that support the findings of this study are available from the corresponding author upon reasonable request.
