# Supplementary material for: Multi‐Scale Bionic Structure Constructs Biomass Flame‐Retardant Thermal Insulation Foam Material
Source: Adv Sci (Weinh). 2026 Aug 11:e77015. Online ahead of print. doi: 10.1002/advs.77015 (PMC13460411; doi:10.1002/advs.77015)
Supplement: Supplementary file 1 [file ADVS-9999-e77015-s002.docx]

**SUPPORTING INFORMATION**

**Multi-scale Bionic Structure Constructs Biomass Flame-Retardant Thermal Insulation Foam Material**

Jianming Liao^a, #^, Lijun Fan^a, c, #^, Yunyuan Dong^a, #^, Xuyu Bao^a^, Xiaobin Chen^a, *^, Qifu Zheng^a^, Guanqing Zhang^a^, Shen Wang^a^, Wei Chen^d^, Min Zhang^a, *^, Shuaiming He^b, *^

^a^ College of Chemical and Material Engineering, Quzhou University, Quzhou 324000, China.

^b^ State Key Laboratory of Woody Oil Resource Utilization, College of Materials and Energy, Central South University of Forestry and Technology, Changsha, 410000, China.

^c^ College of Chemical Engineering, Zhejiang University of Technology, Hangzhou 310000, China.

^d^ School of Pharmaceutical and Chemical Engineering, Taizhou University, Jiaojiang 318000, China.

*Corresponding author.

Email: [xiaobinchen@qzc.edu.cn](mailto:xiaobinchen@qzc.edu.cn) (X.C.); [mzhang.chemmater@qzc.edu.cn](mailto:mzhang.chemmater@qzc.edu.cn) (M. Z.); [minghe@csuft.edu.cn](mailto:minghe@csuft.edu.cn) (S.H.)

^#^ J.L., L.F., and Y.D. contributed equally to this work.

**Keyword:** Cellulose; flame retardancy; thermal insulation; composites; biodegradability

**Supplementary Note 1:** DFT simulation

To study the intermolecular interactions among cellulose‑DA‑BT/cellulose‑cellulose, the monomer structures of cellulose, dopamine and montmorillonite were built using the Gaussian View program for periodic supercells. All monomer structures were optimized at the B3LYP/def2‑TZVP level using the ORCA program. The optimized periodic supercells of cellobiose, dopamine and montmorillonite (such as Montmorillonite‑Na) were combined into cluster structures according to the molar ratio using the Molclus program. Then, the MOPAC software was used to perform semi‑empirical optimization at the PM6‑DH+ level. The optimized structures were sorted by energy using Molclus, and the five groups with the lowest energy and most stable structures were selected for DFT calculations. The optimized structures were further optimized at the B3LYP‑D3/def2‑TZVP level using the ORCA program. The structure with the lowest energy was selected for single‑point energy calculation, which was also performed at the ωB97M‑V/ma‑def2‑TZVP level using the ORCA program. After full geometry relaxation at the B3LYP‑D3/ma‑def2‑TZVP level, the models exhibit physically reasonable intermolecular distances (e.g., typical O–H···O hydrogen‑bond distances of 1.5–2.5 Å), with no unphysical steric repulsions, confirming the structural reliability of the selected configurations (see Supplementary Figs. S6 and S7). The binding energies were then derived from the single‑point energies. For the two‑body interactions (cellulose–cellulose, cellulose–DA, and DA–BT), the standard counterpoise procedure was applied to correct for basis‑set superposition error (BSSE). For the ternary cellulose–DA–BT system, we report the uncorrected total binding energy, based on two solid theoretical considerations: first, a rigorous many‑body CP correction for the ternary complex is mathematically ambiguous owing to the intricate fragmentation scheme and computationally prohibitive; second, and more importantly, all our energy calculations employ the large triple‑zeta basis set with diffuse functions (ma‑def2‑TZVP), which is already near the complete‑basis‑set limit, so the residual BSSE is negligible and does not affect our qualitative or quantitative conclusions regarding the strong interfacial interactions. In Supplementary Note 1, we provide a detailed comparison of our computed interaction energies with literature data for similar hydrogen‑bonded and electrostatic systems, showing that our values are consistent with the typical range (generally –20 to –300 kJ/mol) and with reported results for dopamine‑modified mineral surfaces and cellulose composites. We also describe the full binding‑energy calculation procedure, including all structural parameters and convergence criteria, to ensure reproducibility.

**Supplementary Note 2:** Simulation of Energy-Efficient Cooling

To evaluate the energy‑saving cooling effect of the CBF foam, a building energy simulation was conducted using the open‑source software EnergyPlus (version 23.1.0). The building model was constructed using SketchUp (Professional Edition 2021). In the simulation, the baseline exterior wall was defined as a conventional residential wall representative of the hot‑summer and warm‑winter climate zone in China, consisting of a 20 mm cement mortar layer, a 200 mm masonry block layer, and a 20 mm cement mortar layer, with a heat transfer coefficient of U = 0.857 W m⁻² K⁻¹ based on the Design Standard for Energy Efficiency of Public Buildings GB 50189‑2015. This configuration served as the reference case for typical thermal performance. For the CBF‑filled wall, a 40 mm CBF insulation layer was added to the baseline assembly, resulting in a wall composition of 20 mm cement mortar + 200 mm masonry block + 40 mm CBF layer + 20 mm cement mortar, with a reduced U value of 0.549 W m⁻² K⁻¹.

The cooling energy consumption of the building model without the CBF foam was first calculated using the baseline wall. Then, the CBF foam was placed as the building filler material (i.e., the CBF‑filled wall) and the cooling energy consumption was recalculated under identical building model, operating schedule, indoor temperature settings, and climatic boundary conditions. Based on the climate data downloaded from the EnergyPlus website (<https://energyplus.net/weather>), the cooling energy consumption of building models with and without CBF foam in different cities in China was compared. The simulation period covered 365 consecutive cooling days, and the DOE‑2 algorithm (Conduction Transfer Function method) was employed for the thermal calculations.

**Supplementary Note 3:** Bentonite (A colloidal clay that consists primarily of montmorillonite.) is generally regarded as a mineral filler with low toxicity and relatively low environmental risks^[1]^.

**Supplementary Note 4:** Biodegradability testing.

The ultimate aerobic biodegradability of materials under controlled composting conditions is determined by measuring carbon dioxide release, in accordance with the GB/T 19277.1-2011 standard. The composting degradation test was conducted in a 2.5‑L quartz glass container. The inoculum was obtained from matured and fermented organic fertilizer prepared through impurity removal and screening, with a pH of 8.25, a moisture content of 47.92%, a total dry solids content of 52.08%, and a volatile solids content accounting for 14.45% of the wet solids. On a dry weight basis, the ratio of inoculum to test material was 6:1. Microcrystalline cellulose was used as the reference material. The test mixture was aerated with carbon dioxide-free air, using the sample as the organic carbon source. The biodegradation rate was determined by measuring the amount of carbon dioxide produced. The average biodegradation rate was calculated based on the total carbon dioxide released at the end of the test and the mass loss of volatile solids. The biodegradability of the designed biomass foam was further evaluated using the soil burial method.

**Supplementary Note 5:** Life Cycle Assessment (LCA) Analysis

This study aims to assess the environmental impact of producing 1 kilogram of CBF foam using a life cycle assessment (LCA) analysis with Simapro 10.1, in accordance with the ISO 14040 standard. The assessment adopted the midpoint (H) V1.07 / World (2010) H method in ReCiPe 2016, which covers 14 environmental impact categories, including climate change, land acidification, freshwater consumption, fossil resource consumption, and human toxicity. For the life cycle inventory, the electricity supply was modeled using the specific dataset “Electricity, low voltage {CN-CSG}” from the Ecoinvent 3 database (cut‑off, version 1.3.0). This dataset is geographically representative of the China Southern Power Grid (CSG) region and reflects the average low‑voltage electricity mix for the period 2021–2023. It is important to note that this dataset does not merely represent generic national grid data; it incorporates grid transmission and distribution losses specifically associated with the step‑down transformation from medium to low voltage (below 1 kV), based on IEA World Energy Statistics. The underlying infrastructure (transformers below 2.5 MVA) and the regional generation mix of the CSG are inherently included in the inventory. Therefore, the geographical boundary of this study is explicitly limited to the CSG region, and the results are contingent upon this specific electricity generation portfolio. For the end‑of‑life stage, all wood‑based components are assumed to undergo 100% landfilling via uncontrolled open dump, following the Ecoinvent waste wood treatment model. The inventory parameters for this disposal scenario include: gross heating value of 15.36 MJ/kg and net heating value of 13.99 MJ/kg; elemental composition (C: 401.470 g/kg, H: 50.163 g/kg, O: 372.180 g/kg, N: 0.987 g/kg, S: 0.126 g/kg, Cl: 0.331 g/kg, F: 0.021 g/kg, P: 0.109 g/kg, along with various trace metals as detailed in the Supporting Information); 100% biogenic carbon content in the waste; zero recyclability for Fe, Al, and Cu; overall degradability of 1.5% within 100 years; and a long‑term residual waste mass of 103.3 mg remaining in the landfill even after the long‑term period.

**Supplement Figures**

**Fig. S1**. Characterization of nanocellulose and its role in foam stabilization. (a) Schematic illustration of nanocellulose, highlighting its high-aspect-ratio fibrillar structure that serves as a reinforcing and interfacial stabilizing agent in the CBF foam matrix. (b) Atomic force microscopy (AFM) height image of nanocellulose, showing individual fibrils with a height of approximately 6.8 nm and a lateral distribution within 200 nm. The uniform nanoscale dimensions and surface topography confirm its ability to effectively adsorb at the air–liquid interface during foaming, thereby stabilizing bubbles and promoting the formation of a fine, homogeneous porous structure in the final CBF foam^[2]^.

**Fig. S2**. In-situ observation of wet-foam evolution. (a) Optical micrograph of the initial wet foam, showing fine bubbles dispersed among pulp fibers shortly after mechanical foaming. (b) Image captured 8 minutes later, illustrating bubble coalescence and growth, driven by interfacial rearrangement facilitated by nanocellulose and dopamine adhesion. Stabilized wet foam after standing, where bubble size becomes uniform and the structure is maintained by the combined action of bentonite-enhanced network strength and nanocellulose-mediated interface retention, leading to the homogeneous porous morphology crucial for thermal insulation and flame retardancy in the final CBF foam.

**Fig. S3**. SEM images of cellulose-based foams. (a) SEM image of CF, which serves as the structural skeleton. (b) SEM image of CBF-0, in which the fibers are uniformly coated with polydopamine (PDA), resulting in a smoother surface that promotes interfacial adhesion and facilitates subsequent bentonite loading. (c) The SEM image of CBF-4 reveals that part of the BT on the PDA-coated fibers exhibits increased interlayer spacing. (d) SEM image of CBF‑P0, showing that only a small amount of BT is attached to the fibers, further demonstrating the adhesive role of PDA.

**Fig. S4.** Elemental analysis of cellulose-based foams. (a) Corresponding EDS spectrum of CBF‑4 confirms the presence and distribution of bentonite‑derived elements (such as Si, Mg, Al), verifying the successful incorporation of BT and its role in improving thermal and flame‑resistant performance. (b) In contrast, the EDS spectrum of CBF‑P0 shows that the intensities of Si, Mg and Al bentonite‑related elements are substantially reduced compared to those of CBF‑4, further confirming that the absence of PDA leads to poor BT adhesion on the fiber surface.

**Fig. S5**. Multiscale foam structure. (a) Schematic of the multi‑stage processing: cellulose fibers from wood pulp are mechanically refined into a multi‑scale network and then surface‑treated with dopamine, which promotes strong interfacial bonding and enables uniform integration with bentonite and nanocellulose during foaming. (b) The electronic photograph of the original cellulose material shows the sustainable supporting structure inside the foam material. (c) Bentonite (BT) mineral particles exhibiting a layered silicate structure; when exfoliated and dispersed in the foam matrix, these layers create a physical barrier that enhances thermal insulation and flame retardancy. (d) Nanocellulose suspension emphasizing its high‑aspect‑ratio nanofibrils, which effectively stabilize bubble interfaces during foaming and reinforce the pore walls, contributing to the foam’s mechanical integrity. (e) Photograph of the resulting CBF foam, showcasing its lightweight, hierarchically porous morphology achieved through the synergistic combination of pulp, dopamine, bentonite and nanocellulose—a design that collectively delivers improved thermal management and flame‑retardant performance for building‑insulation applications.

**Fig. S6**. The DFT simulation results of Cellulose–DA–BT. (a) DES illustrates significant charge redistribution at the interface, thereby ensuring structural integrity during thermal exposure. (b) IRI iso-surfaces reveal extensive non-covalent interaction networks, wherein van der Waals forces and hydrogen bonds work synergistically to stabilize the foam and reinforce its cohesion. (c‑d) OPT and AIM analyses reveal the interfacial bonding and electronic interactions. The OPT plot shows pronounced orbital overlap, indicating strong electronic coupling. AIM analysis identifies specific bonds: BT links to cellulose via hydrogen bonds (O–H···O, 2.35 Å) and ion‑dipole interactions (H···Na⁺, 2.15 Å). Dopamine acts as a bridge, bonding with cellulose (O–H···O, 2.54 Å) and electrostatically attracting BT. This “electrostatic fixation”, along with van der Waals forces, forms a tight hybrid interface, key to the enhanced mechanical, thermal and flame‑retardant properties of CBF‑4.

**Fig. S7**. The DFT simulation results of cellulose-cellulose. (a)DES; (b)IRI; (c)OPT.(d)AIM. Characterizing the electrostatic potential energy landscape underlying the intermolecular interactions of cellulose, we find that these interactions are predominantly noncovalent, and that the electrostatic potential varies markedly with the extent of interfacial contact. Regions of negative potential are associated with attractive interactions—principally hydrogen bonding and van der Waals force—that drive molecular aggregation and ordering; by contrast, regions of positive potential are associated with electrostatic repulsion, which becomes especially pronounced under spatial confinement.

**Fig. S8** The cross-sectional images of Micro-CT showing the distinct bright pore walls in CBF (a) formed by the high electron density of embedded BT nanosheets, while the CF (b) exhibits a uniform grayscale.

**Fig. S9**. (a)The Micro-CT reconstructed images and (b) cross-sectional view of CBF-0 reveal a distinct morphological evolution compared to CF, which typically exhibits inherent fibrillar roughness. This transformation is attributed to the in-situ polymerization of dopamine, which forms a relatively smooth continuous polydopamine (PDA) film coating the fiber surfaces. This coating effect, resulting from the water-insoluble nature of PDA, effectively locks the cellulose structure and prevents its disintegration upon water exposure.

**Fig. S10**. Raman spectral analysis of carbonized structures. (a) Peak‑fitting of the D and G bands for BT. The relatively low I_D_/I_G_ ratio indicates that the bentonite‑reinforced pulp retains a graphitic‑like microcrystalline arrangement after carbonization, with limited structural defects. (b) Peak‑fitting of the D and G bands for CBF. The significantly higher I_D_/I_G_ ratio reveals that dopamine, upon thermal conversion, forms a nitrogen‑rich amorphous carbon layer which covers or disrupts the originally ordered graphitic domains. Concurrently, the foaming process introduces additional edge defects, while the incorporation of PDA further increases structural disorder—a characteristic signature of heteroatom doping and enhanced amorphous carbon content. These structural changes contribute to the formation of a more continuous and resilient char layer during combustion, thereby improving the flame‑retardant and thermal‑barrier performance of the final CBF‑4 foam.

**Fig. S11**. (a) FT-IR confirms dopamine/bentonite incorporation and cross-linked phase formation. (b) Enlarged view in the region 400–1500 cm⁻¹. The appearance of the Al–O–Si absorption band in CBF‑4 further confirms the successful loading of montmorillonite (the main component of BT) onto the cellulose fibers. These spectral changes collectively verify the effective hybridization of bentonite with the bio‑based foam network, which enhances the thermal‑barrier stability and flame‑retardant performance of the final CBF‑4 material.

**Fig. S12.** Characterization of CBF-P0 and CBF-4 by multiple techniques. (a) FTIR reveals a red shift and broadening of the O–H band in CBF-4, confirming the formation of a hydrogen-bond network between PDA and cellulose. (b) ¹³C NMR shows unchanged chemical shifts but a marked attenuation of the amorphous C4 signal at ~88.9 ppm, indicating that PDA preferentially anchors at amorphous regions via hydrogen bonding and restricts chain mobility. (c) XRD demonstrates that the undulating profile in the low-angle region (2𝜃 = 10°–22°) disappears in CBF-4, suggesting disruption of short-range ordered stacking. (d) O 1s XPS peak broadening reflects altered oxygen electron density arising from hydrogen-bond formation. (e) In the C 1s spectra, the C–O component weakens (consistent with hydroxyl participation), whereas the C–C/C–H and C=O peaks intensify. These changes are attributed to the PDA aromatic skeleton and quinone structures, confirming PDA loading and interfacial bonding. (f) Si 2p peak broadening indicates interaction between PDA and Si–OH groups on the BT surface, promoting uniform BT dispersion.

**Fig. S13**. XPS survey spectrum confirming the coexistence of characteristic elements: Si and Al from bentonite (BT) and N from polydopamine (PDA), which verifies the successful integration of both components into the CBF‑4 composite.

**Fig. S14**. XPS analysis of BT, CF and CBF‑4. (a) O 1s XPS shows increased Si-O bonds correlating with improved insulation and flame retardancy. (b) High resolution C 1s spectrum CBF 4 shows a distinct C-N peak at 286.5 eV from PDA, indicating the presence of polydopamine and its interfacial interaction with BT, which enhances the thermal stability and flame retardancy of the bio - foam. (c‑d) High‑resolution Si 2p and Al 2p spectra. For CBF‑4, the Si–O peak (≈102.2 eV in BT) shifts positively to ≈102.5 eV, and the Al–O peak (≈74.8 eV) shifts to ≈75.1 eV. These consistent shifts reflect electron‑cloud redistribution driven by extensive hydrogen‑bonding between BT surface –OH groups and the –OH/–NH₂ groups of cellulose/PDA, which strengthens the interface and improves the foam’s mechanical integrity and thermal‑barrier performance.

**Fig. S15**. (a) The compression stress-strain curves and (b) inter-fiber bond strength curve of CF, CBF-0 and CBF-4. The maximum compressive strength of CBF-4 is approximately 123.6 kPa, which is about three times that of CF. Moreover, CBF-4 has the strongest adhesion and, under the same displacement, it can withstand the greatest force. It is indicated that bentonite and dopamine enhance the binding force between the fibers.

**Fig. S16**. Comparison of compressive modulus (a) and specific modulus (b) of CF, CBF-0 and CBF-4. The modulus of CBF-4 (4.19) is significantly higher than that of the other two groups. Compared with CF and CBF-0, it has increased by approximately 2.6 times. The adhesion and reactivity of dopamine may tightly bind the bentonite nanosheets to the cellulose fibers, forming a three-dimensional reinforced network, enabling the foam structure to possess high stiffness even at a low density.

**Fig. S17**. Infrared thermal imaging of CBF under heating. The thermal image records the surface‑temperature distribution of the CBF sample and the underlying heating plate during steady‑state heating. This contrast clearly demonstrates the effective thermal‑insulation capability of the foam, which originates from its hierarchical porous structure reinforced by bentonite and dopamine. The bentonite‑enhanced matrix impedes heat conduction, while the fine, uniform pores stabilized by nanocellulose and dopamine reduce radiative and convective heat transfer.

**Fig. S18**. Thermal‑response test using petals placed on CBF‑0. A fresh petal was placed on the surface of the CBF‑0 foam and subjected to heating. After 60 s, the petal showed clear charring, indicating that the foam surface reached a temperature high enough to induce rapid pyrolysis of the organic tissue. This result highlights the limited thermal‑barrier performance of the dopamine‑only formulation (CBF‑0), in which the absence of bentonite leads to insufficient insulation and faster heat transfer through the porous matrix. The charring of the petal visually demonstrates the need for synergistic enhancement with bentonite as implemented in CBF‑4 to form a more effective heat‑blocking structure for flame‑retardant and insulation applications.

**Fig. S19**. Thermal stability and expansion behavior of CBF foam. (a) Comparison of foam morphology at 25 °C and after exposure to 200 °C for 5 min. The CBF foam retains its structural integrity without noticeable softening or deformation, demonstrating excellent thermal stability imparted by the synergistic bentonite‑dopamine‑nanocellulose network. (b) Comparison of thermal expansion coefficients. The CBF foam exhibits a significantly lower expansion coefficient than several petroleum‑based plastics (e.g., PP, PE). After 5 min at 200 °C, those commercial plastics have visibly softened and deformed, whereas the CBF maintains its shape and dimensional stability, highlighting its suitability for high‑temperature insulation applications^[3]^.

**Fig. S20.** Thermal conductivity measurements of different samples, showing that CBF‑4 exhibits the lowest value, attributed to its hierarchical pores and the synergistic effect of bentonite and dopamine.

**Fig. S21**. Water‑resistance performance of CBF. (a) Image of the water resistance test. (b) Contact angle measurement: an increased angle confirms hydrophobic modification via grafting of alkyl chains, enhancing moisture resistance while preserving the porous structure for thermal insulation. (c) Enhanced C–H stretching peaks (2800–3000 cm⁻¹) in the FTIR spectra confirm grafting. (d) UL‑94 burning tests: the PDMS‑coated foam achieves V‑0 rating, identical to uncoated foam, confirming that flame retardancy is maintained.

**Fig. S22**. Schematic diagram of a sample building model for Energy Plus simulation. Use this model to conduct energy consumption simulation for cooling in common residential buildings.

**Fig. S23**. Photos of flame resistance of CBF-0. When exposed to a flame at approximately 1300 ℃, cellulose will rapidly catch fire and burn almost completely within one minute. Eventually, only a small number of carbonized fibers will remain.

**Fig. S24**. CBF horizontal and vertical combustion experiment. The combustion characteristics of CBF foam were tested in both horizontal and vertical directions. CF and CBF-0 foam showed obvious flammability, with total combustion times of 33 seconds and 23 seconds, 25 seconds and 24 seconds respectively. During the combustion process, the foam structure collapsed and shrank. In contrast, CBF-4 foam had self-extinguishing properties, resulting in a gradual shortening of the combustion time.

**Fig. S25**. The LOI value of CBF. LOI (Limit of Ignition) is an indicator that measures the minimum oxygen concentration required for a material to sustain combustion in a nitrogen-oxygen mixture. LOI < 21%: flammable (the oxygen content in the air is approximately 21%); LOI > 28%: non-flammable, will extinguish by itself; LOI > 35%: high flame retardancy^[4]^. CF has almost no flame retardancy and is highly flammable (easily ignitable). CBF-1: LOI = 29.4%, reaching the non-flammable level, indicating that the addition of bentonite and dopamine significantly improves the flame retardancy. CBF-4: LOI = 44.8%, has excellent flame retardancy, indicating that the synergistic effect of this ratio is the strongest.

**Fig. S26**. Analysis of flame‑retardancy using the cone calorimeter. (a) Comparison of peak heat‑release rate (PHRR). CBF‑4 exhibits a significantly reduced PHRR (60.3 kW m⁻²) due to the barrier effect of bentonite‑reinforced char and dopamine‑enhanced carbon‑layer integrity, which effectively slows down heat transfer.
(b) Effective combustion heat (EHC) and corresponding optical photos of CF, CBF‑0, and CBF‑4. The low EHC of CBF‑4 confirms that the synergistic action of bentonite and dopamine suppresses the combustion efficiency by promoting char formation and releasing non‑flammable gases. (c) Smoke‑release rate, which drops nearly to zero after 50 s, indicating that the composite foam rapidly forms an insulating char that restricts smoke production. (d) CO₂‑release rate, reduced by 53.4 % in CBF‑4.

**Fig. S27**. After the cone-shaped combustion test was completed, only a small amount of residue remained for CF and CBF-0, while the surface of CBF-4 was covered with a layer of carbon

**Fig. S28**. The TGA curve shows that while CF undergoes sharp decomposition at 350°C, the addition of bentonite and dopamine shifts the degradation peak to higher temperatures in CBF-0 and CBF-4. Notably, CBF-4 exhibits a broader and lowered peak, indicating delayed thermal decomposition due to a synergistic thermal barrier effect from bentonite’s layered structure and dopamine-derived carbon layers, which enhance the material’s thermal stability and char-forming ability.

**Fig. S29**. TG-IR spectra of CF (a), CBF-0 (b) and CBF-4 (c). TG-FTIR analysis reveals a shift toward condensed-phase decomposition, shown by reduced organic bands and a higher CO₂/H₂O ratio. PDA strengthens fiber interlayer bonding via hydrogen networks, creating a tortuous barrier that slows heat/mass transfer and raises the main TG decomposition temperature. The modification fosters an organic-inorganic coupled char, where PDA’s carbon-rich aromatic structure interlocks with silicate layers to form a dense, oxidation-resistant interface. CBF's flame retardancy results from three synergistic mechanisms: interfacial oxidation-complexation on cellulose, PDA’s gas-phase radical scavenging, and silicate-promoted condensed-phase charring. Together, these improve thermal stability and suppress flammability while maintaining the foam’s low density and porosity.

**Fig. S30**. High-resolution XPS spectra of (a) C 1s and (b) O 1s for CBF-0 are presented. In the C 1s spectrum, the sample exhibits the emergence of a C=N peak (~285.8 eV), characteristic of polydopamine structures. In the O 1s spectrum, a significant enhancement of the C–O–C/C–O–H component is observed. These changes provide direct evidence for the successful grafting of polydopamine onto the cellulose fiber surface and the reinforcement of the interfacial hydrogen-bonding network.

**Fig. S31**. Structural analysis of combustion residues. (a) FT‑IR spectra of residues after combustion. CBF‑4 exhibits strong Si–O–Si peaks at ~1000 and ~447 cm⁻¹, confirming that bentonite forms a robust, “ceramic‑like” inorganic framework within the char. This silicon‑oxygen network significantly enhances thermal stability and flame retardancy. In contrast, CF and CBF‑0 lack these inorganic signals, as they produce only organic carbonaceous residues. (b) XRD patterns of the residues. CBF‑4 shows a distinct low‑angle peak at ~6°–8°, indicating that the layered silicate structure of bentonite is partially retained after combustion, forming an ordered inorganic barrier. This “labyrinth‑effect” framework hinders heat and mass transfer, while the organic‑derived peaks (~11°–13°) disappear due to the disruption of molecular ordering by the clay layers. CF and CBF‑0 retain only weak organic diffraction features, which lack the thermal stability of the inorganic framework. The preserved inorganic layered structure in CBF‑4 is key to its superior flame‑retardant and insulation performance.

**Fig. S32**. SEM images of residues after combustion are shown. In CF (a, b), the residue exhibits a collapsed and fragmented network with large cracks and little coherent structure, indicating extensive thermal degradation and poor char integrity. In CBF-0 (c, d), the char forms a denser but still discontinuous carbon layer containing some bubble-like pores; the dopamine-derived carbon provides limited consolidation but does not establish a sufficiently robust framework to prevent structural collapse. In CBF-4 (e, f), a continuous and compact char layer with a consolidated ceramic-like morphology is observed. The layered silicate framework derived from bentonite remains intact, forming a coherent barrier that effectively insulates the underlying material and restricts flame propagation.

**Fig. S33**. Differential electron-density (DES) mapping of CBF-4 reveals a uniform electron-density distribution throughout the char residue, confirming the homogeneous integration of inorganic bentonite layers within the carbonized organic matrix. This dense hybrid interface enhances thermal stability and contributes to the superior flame-retardant performance of CBF-4.

**Fig. S34.** Appearance evolution and optical performance of CBF foams during accelerated UV aging. CBF foam specimens were exposed to xenon-arc lamp weathering according to ASTM G155. Test conditions: irradiance of 0.35 W/(m²·nm) at 340 nm, black-panel temperature of 60 °C, and total exposure duration of 168 h (7 days). Photographs were taken at 0, 24, 48, 72, 96, 120, 144 and 168 h to document macroscopic appearance changes (a). Surface reflectance spectra were recorded at each time interval using a UV-Vis spectrophotometer (b). No significant discoloration or structural degradation was observed over the entire aging period, confirming the excellent UV resistance of the CBF foams.

**Fig. S35**. Different raw materials can all successfully be used to produce CBF. (a) bamboo pulp; (b) Broadleaf wood pulp; (c) hemp pulp; (d) waste paper pulp.

**Fig. S36**. Microscopic images of coniferous wood pulp fibers (a) before processing and (b) after processing. After pre-treatment, it can be observed that the fibers have been effectively separated and fibrillated, with many fine fibers exposed on the surface. (This pretreatment process involved mechanical disintegration and pulping to separate and fibrillate the fibers, followed by centrifugal dewatering to adjust the moisture content, ultimately yielding a fiber slurry with a freeness of 440 mL CSF.)

**Fig. S37**. A comparison was made before and after the treatment of the fibers. (a) Fibers weighted by length, (b) Fiber width, (c) Weighted ratio of fine fibers. The proportion of fine fibers in the pre-treated fibers is higher (49.43%), indicating that these fibers are more refined. The moderately pulped coniferous long fiber pulp is a more common and ideal choice. It can provide sufficient long fibers to form the framework, and through the pulping process, it can produce an appropriate number of fine fibers, thereby optimizing the hydrogen bond combination and network strength between the fibers.

**Supplement Tables**

**Table S1:** Comparison of thermal conductivity and coefficient of thermal expansion data for commercial polymer foams and bio-based materials.

| Materials | Thermal insulation (W/mK) | Ref number | Thermal expansivity (1×10^−6^K^−1^) | Ref number |
| --- | --- | --- | --- | --- |
| PF | 0.058 | ^[5]^ | 16 | ^[6]^ |
| PU | 0.05 | ^[7]^ | 100-200 | ^[6]^ |
| PI | 0.0535 | ^[8]^ | 30-60 | ^[6]^ |
| CNFs thermo-mechanical structural materials | 0.0486 | ^[9]^ | 65.1 | ^[10]^ |
| Biomimetic structural nanocellulose materials | 0.083 | ^[11]^ | 5 | ^[12]^ |
| TCNF aerogel in a hollow fiber made from cellulose acetate | 0.07 | ^[13]^ | 80 | ^[6]^ |
| This work | 0.048 |  | 0.63 |  |

Because studies on commercial polymer foams and biobased materials that possess both thermal conductivity and thermal expansion coefficient data are extremely rare, this study adopted a joint citation strategy. For each foam type, thermal conductivity and thermal expansion coefficient data were collected from different high-quality literature sources, provided that the biobased source type, experimental temperature range, and characterization methods were comparable.

**Table S2:** The densities of CBF foams at different proportions.

| Materials | Density (g/cm^3^) |
| --- | --- |
| CF | 0.017±0.0030 |
| CBF-0 | 0.021±0.0005 |
| CBF-1 | 0.026±0.0015 |
| CBF-2 | 0.028±0.0010 |
| CBF-3 | 0.025±0.0010 |
| CBF-4 | 0.028±0.0020 |

**Table S3:** Annual building model simulation data table of cooling energy consumption in different cities.

| City | Without CBF（MJ/m^2^） | With CBF  （MJ/m^2^） | Energy saving  (%) |
| --- | --- | --- | --- |
| Macao | 427.1346 | 145.6182 | 65.67% |
| Beijing | 264.6346 | 81.7218 | 69.12% |
| Changchun | 123.0563 | 37.3338 | 69.66% |
| Changsha | 301.6462 | 97.16904 | 67.79% |
| Chengdu | 208.8212 | 62.39448 | 70.12% |
| Chongqing | 286.8523 | 90.0828 | 68.60% |
| Fuzhou | 335.5348 | 113.3928 | 66.21% |
| Guangzhou | 356.1516 | 121.8416 | 65.79% |
| Guiyang | 169.0301 | 51.9552 | 69.26% |
| Harbin | 131.8712 | 38.24316 | 71.00% |
| Haikou | 432.7106 | 149.1966 | 65.52% |
| Hangzhou | 284.1786 | 91.78632 | 67.70% |
| Hefei | 286.9722 | 94.96152 | 66.91% |
| Hohhot | 204.2453 | 50.39352 | 75.33% |
| Jinan | 270.5818 | 87.27984 | 67.74% |
| Kunming | 170.5799 | 42.0462 | 75.35% |
| Lanzhou | 212.7488 | 50.59404 | 76.22% |
| Lassa | 140.4994 | 21.24468 | 84.88% |
| Nanchang | 351.4705 | 112.1818 | 68.08% |
| Nanjing | 276.0206 | 89.97264 | 67.40% |
| Nanning | 406.7611 | 134.1439 | 67.02% |
| Shanghai | 259.8941 | 85.8906 | 66.95% |
| Shenyang | 180.6739 | 54.44424 | 69.87% |
| Shijiazhuang | 295.0506 | 90.87768 | 69.20% |
| Taiwan | 394.3285 | 138.771 | 64.81% |
| Taiyuan | 213.2957 | 60.0426 | 71.85% |
| Tianjin | 262.9818 | 80.39772 | 69.43% |
| Wuhan | 357.8886 | 112.5209 | 68.56% |
| Urumqi | 212.791 | 56.36952 | 73.51% |
| Xi'an | 253.7172 | 76.66344 | 69.78% |
| Hong Kong | 364.9072 | 125.2692 | 65.67% |
| Xining | 133.1323 | 22.93524 | 82.77% |
| Yinchuan | 193.0014 | 53.3106 | 72.38% |
| Zhengzhou | 299.3987 | 95.02092 | 68.26% |

**Table S4.** Comparison of thermal performance between CBF foam (this work) and commercial insulation materials reported in literature.

| Parameter | λ (W/m·K) | U‑value (W/m²·K) | U‑value reduction | Energy saving rate | Study focus | Data source |
| --- | --- | --- | --- | --- | --- | --- |
| Uninsulated | / | 0.857 | / | / | / | GB 50189‑2015 |
| + XPS (40 mm) | 0.030–0.035 | 0.400–0.439^2^ | 48.8–53.3% | Not reported¹ | Heating / Cold climate | GB/T 10801.2‑2018; ^[14][15][16]^ |
| + Rock wool (40 mm) | 0.040 | 0.461^2^ | 46.2% | Not reported¹ | Heating / Cold climate | GB/T 25975‑2018; ^[14][15]^ |
| + CBF foam (40 mm) | 0.0488 (measured) | 0.549 | 35.9% | ~70% (cooling, dynamic) | Cooling / Hot‑summer & warm‑winter (China) | This work |

1. Literature studies focus on heating‑season performance (HDD method); cooling‑season energy saving rates are not reported.

2. The U-values of EPS/XPS/rock wool are calculated based on the thermal conductivity limits stipulated in national standards, using the formula in GB 50176 2016.

**Table S5:** Input and output conditions and energy consumption data for the laboratory‑scale batch preparation of 1 kg CBF foam.

|  | **Input/Output** | **Quantity** | **Unity** |
| --- | --- | --- | --- |
| **Step 1: Prepare pulp and buffer solution** | **Input** | | |
|  | Softwood pulp | 1.00 | kg |
|  | Tris HCl | 62.8 | g |
|  | NaOH | 8.00 | g |
|  | Water | 103.89 | kg |
|  | Energy (beating machine) | 0.000375^a^ | kWh |
|  | **Output** | | |
|  | Pulp | 1.00 | kg |
|  | Tris-HCl Buffer solution | 40.00 | kg |
| **Step 2: Making the slurry** | **Input** | | |
|  | Pulp | 0.50 | kg |
|  | Dopamine HCl | 50.00 | g |
|  | Tris-HCl buffer solution | 40.00 | m^3^ |
|  | Energy (beater) | 0.043^b^ | kWh |
|  | **Output** | | |
|  | Pulp oxidation | 0.55 | kg |
| **Step 3: Foaming drying** | **Input** | | |
|  | Pulp oxidation | 0.55 | kg |
|  | Bentonite | 0.50 | kg |
|  | SDS | 20.00 | g |
|  | TEMPO-CNF | 30.00 | g |
|  | Energy (dispersion and drying) | 2.543^c^ | kWh |
|  | **Output** | | |
|  | Liquid including Tris-HCl Buffer solution and Dopamine HCl (solid content20%)^1^ | 15.00 | kg |
|  | Water stream | 5.00 | kg |
|  | CBF | 1.00 | kg |

(a) The power required for pulping without bleaching to increase the degree of pulping (0.75-1.50 kWh/t). In this calculation, the pulping power is set at 0.75 kWh/t, the pulping time is 0.50 h, and the pulp used is 0.001 t (the standard amount used by the pulping machine). The power consumption is 0.75 kWh/t×0.50 h×0.001 t≈0.000375 kWh.

(b) According to the recommended average stirring power per unit volume of liquid (solid organic matter suspension: 0.18-0.29 kWh/m^3^), stir for 12 h, and the volume of the dissolved liquid is approximately 0.02 m^3^. The power consumption is 0.180933 kWh/m^3^×12 h×0.02 m^3^≈0.043 kWh.

(c) (1) Flooding process: Take the flooding machine power as 0.75 kW (common motor of laboratory flooding/dispersion machines is 0.75-1.5 kW), stir at 3000 rpm (speed 3000 r/min), the maximum capacity of the fiber standard disintegrator slurry is 2 L, the power consumption is 3000/3000 r/min/60×0.75 kW×10×2 L≈0.125 kWh

(2) Drying process: (The specific heat capacity of water is 4200 J/kg℃). (1 Heating stage: The energy consumption required to heat water from 25℃ to 60℃ is 35×5×4200/3600000=0.204 kWh. (2 Insulation stage: The temperature of the air in the laboratory oven decreases by approximately 1℃ per minute. The heat loss in 6 hours is: 5×4200×1/3600000× (6×60) =2.1 kWh. Therefore, the total energy consumption of the foam in the oven is 0.2042.1=2.214 kWh.

1: Ignoring the influence of SDS, the total mass after water addition = solid mass / solid content=1 kg/0.20=5 kg.

Note: The energy consumption values were obtained from laboratory‑scale batch equipment and are provided only as internal references for the sample preparation process in this study. They should not be directly extrapolated to industrial‑scale continuous production.

**Table S6:** Absolute characterization results for all 14 impact categories per functional unit (CBF vs. reference materials).

| Impact category | unit | CBF | PP | PE | PS | PU |
| --- | --- | --- | --- | --- | --- | --- |
| Global warming | kg CO_2_ eq | 2.395647 | 3.973375 | 4.39062 | 11.21174 | 7.468717 |
| Stratospheric ozone depletion | kg CFC11 eq | 5.57E-07 | 6.88E-07 | 9.18E-07 | 1.20E-05 | 1.46E-05 |
| Ozone formation, Human health | kg NO_x_ eq | 0.006727 | 0.007886 | 0.010061 | 0.009624 | 0.015572 |
| Fine particulate matter formation | kg PM2.5 eq | 0.003572 | 0.00417 | 0.005229 | 0.005286 | 0.010356 |
| Ozone formation, Terrestrial ecosystems | kg NO_x_ eq | 0.006911 | 0.008781 | 0.01133 | 0.010354 | 0.016727 |
| Terrestrial acidification | kg SO_2_ eq | 0.008455 | 0.008927 | 0.010693 | 0.013573 | 0.022548 |
| Freshwater eutrophication | kg P eq | 0.003892 | 0.006553 | 0.007835 | 0.007394 | 0.008488 |
| Terrestrial ecotoxicity | kg 1,4-DCB | 8.574312 | 27.57829 | 32.57884 | 16.64612 | 100.7849 |
| Freshwater ecotoxicity | kg 1,4-DCB | 0.055432 | 0.287102 | 0.320471 | 0.302184 | 0.562893 |
| Marine ecotoxicity | kg 1,4-DCB | 0.081018 | 0.4132 | 0.464734 | 0.426629 | 0.828028 |
| Human carcinogenic toxicity | kg 1,4-DCB | 0.312395 | 0.650106 | 0.707111 | 0.374144 | 1.270253 |
| Human non-carcinogenic toxicity | kg 1,4-DCB | 1.924036 | 6.050809 | 6.92851 | 9.236153 | 13.13081 |
| Fossil resource scarcity | kg oil eq | 0.586187 | 2.007389 | 2.135041 | 2.132609 | 2.509908 |
| Water consumption | m^3^ | 0.00913 | 0.01836 | 0.045843 | 0.073274 | 0.062453 |

**References**

1. US/ICCA, “SIDS Initial Assessment Profile,” in SIAM, vol. 25 (2007).

2. X. Yang, S. K. Biswas, J. Han, et al., “Surface and Interface Engineering for Nanocellulosic Advanced Materials,” *Advanced Materials* *33*, no. 28 (2021): 2002264, https://doi.org/https://doi.org/10.1002/adma.202002264.

3. J. Hu, Y. Zhang, Y. He, et al., “Transformation of Bamboo: From Multiscale Fibers to Robust and Degradable Cellulose-Based Materials for Plastic Substitution,” *Small* *21*, no. 27 (2025): 1–12, https://doi.org/10.1002/smll.202411339.

4. Y. Lei, Q. N. Chan, L. Xu, E. W. M. Lee, Y. X. Lee, V. Agarwal, G. H. Yeoh, W. Wang, “Smart Retardant Materials for Fire Alarm Systems: Integrating Flame Retardancy and Early Detection Technologies”, *Advanced Composites and Hybrid Materials* (2025) 8:112 https://doi.org/10.1007/s42114-024-01152-6.

5. L. Song, Y. Wu, J. Xue, J. He, and C. Wang, “A High-Temperature Resistant Phosphate-Phenolic Composite Foam with Low Density, High Strength, Flame Retardant and Thermal Insulating Properties,” *Composites Part B: Engineering* *297* (2025): 112294, https://doi.org/https://doi.org/10.1016/j.compositesb.2025.112294.

6. L. Biopolymers, S. Mazurchevici, D. Vaideanu, D. Rapp, and C. Varganici, “Dynamic Mechanical Analysis and Thermal Expansion Of,” *Polymers* *13* (2021): 2953, <https://doi.org/10.3390/polym13172953>.

7. L. Sun, Y. Guo, R. Ou, et al., “Ultrastrong and Thermo-Remoldable Lignin-Based Polyurethane Foam Insulation with Active-Passive Fire Resistance,” *Advanced Functional Materials* *34*, no. 40 (2024): 2405424, https://doi.org/https://doi.org/10.1002/adfm.202405424.

8. H. Zhang, X. Fan, W. Chen, Y. Wang, C. Liu, B. Cui, G. Li, J. Song, D. Zhao, D. Wang, L. Zhao, X. Zhang, H. Xu, C. Chen, “A simple and green strategy for preparing flexible thermoplastic polyimide foams with exceptional mechanical, thermal-insulating properties, and temperature resistance for high-temperature lightweight composite sandwich structures”, *Composites Part B: Engineering* **2022**, *228*, 109405, https://doi.org/https://doi.org/10.1016/j.compositesb.2021.109405.

9. J. M. Liao, P. C. Luan, Y. X. Zhang, L. Chen, L. Y. Huang, L. H. Mo, J. Li, Q. A. Xiong, “A lightweight, biodegradable, and recyclable cellulose-based bio-foam with good mechanical strength and water stability”, *Journal of Environmental Chemical Engineering* **2022**, *10*, https://doi.org/10.1016/j.jece.2022.107788.

10. T. Hirano, K. Mitsuzawa, S. Ishioka, et al., “Anisotropic Thermal Expansion of Transparent Cellulose Nanopapers,” *Frontiers in Chemistry* *8*, no. February (2020): 6–11, https://doi.org/10.3389/fchem.2020.00068.

11. Y. Yang, Y. Chen, Y. Li, et al., “Interfacial Assembly of Nanocellulose Microgels Enhances Thermal Insulation of Pickering Foam,” *International Journal of Biological Macromolecules* *282* (2024): 136796, https://doi.org/https://doi.org/10.1016/j.ijbiomac.2024.136796.

12. Q.-F. Guan, H.-B. Yang, Z.-M. Han, et al., “Lightweight, Tough, and Sustainable Cellulose Nanofiber-Derived Bulk Structural Materials with Low Thermal Expansion Coefficient,” *Science Advances* *6*, no. 18 (2026): eaaz1114, https://doi.org/10.1126/sciadv.aaz1114.

13. V. Apostolopoulou-Kalkavoura, P. Munier, and L. Bergström, “Thermally Insulating Nanocellulose-Based Materials,” *Advanced Materials* *33*, no. 28 (2021): 2001839, https://doi.org/https://doi.org/10.1002/adma.202001839.

14. I. Ahmad Ganaie, O. Gulzar, N. Ali Mir, and M. Afzal, “Dynamic Thermal Performance & Economic Analysis of Insulation Materials in Temperate-Himalayan Regions: A Simulation-Based Approach,” *Energy and Buildings* *353* (2026): 116878, https://doi.org/https://doi.org/10.1016/j.enbuild.2025.116878.

15. A. Aslan, “Effect of Thermal Insulation on Building Energy Efficiency in Turkey,” *Proceedings of the Institution of Civil Engineers - Energy* *175*, no. 3 (2022): 119–139, https://doi.org/10.1680/jener.21.00053.

16. J. Huang, S. Wang, F. Teng, and W. Feng, “Thermal Performance Optimization of Envelope in the Energy-Saving Renovation of Existing Residential Buildings,” *Energy and Buildings* *247* (2021): 111103, https://doi.org/https://doi.org/10.1016/j.enbuild.2021.111103.
